# Association between red cell distribution width/serum albumin ratio and diabetic kidney disease

**DOI:** 10.1111/1753-0407.13575

**Published:** 2024-06-26

**Authors:** Jiaqi Chen, Daguan Zhang, Depu Zhou, Zhijuan Dai, Jie Wang

**Affiliations:** ^1^ Department of Endocrinology Second Affiliated Hospital and Yuying Children's Hospital of Wenzhou Medical University Wenzhou China; ^2^ Department of Gastroenterology First Affiliated Hospital of Wenzhou Medical University Wenzhou China; ^3^ Department of Endocrinology First Affiliated Hospital of Wenzhou Medical University Wenzhou China

**Keywords:** albumin, diabetic kidney disease, red cell distribution width

## Abstract

**Background:**

Previous studies have shown that the red cell distribution width (RDW)/serum albumin ratio (RA) is an integrative and new inflammatory marker. RA is associated with clinical outcomes in a variety of diseases, but the clinical value of RDW/RA in the assessment of diabetic kidney disease (DKD) has not been elucidated. We examined the link between diabetic RA and DKD while controlling for a wide variety of possible confounders.

**Methods:**

Retrospective cohort analysis of the National Health and Nutrition Examination Survey (NHANES: 2009–2018) database from the Second Affiliated Hospital and Yuying Children's Hospital and the Wenzhou Medical University (WMU) database was conducted. Multivariate logistic regression analysis was used to assess the association between RA and DKD.

**Results:**

Overall, 4513 diabetic patients from the NHANES database (*n* = 2839) and the WMU (*n* = 1412) were included in this study; 974 patients were diagnosed with DKD in NHANES and 462 in WMU. In the NHANES cohort, diabetes mellitus (DM) patients with higher RA level had a higher risk of DKD (odds ratio = 1.461, 95% confidence interval: 1.250–1.707, *p* < 0.00001). After adjusting for confounders and propensity score‐matched (PSM) analysis, both shown RA levels were independently linked to DKD (*p*_Adjust_ = 0.00994, *p*_PSM_ = 0.02889). Similar results were also observed in the WMU cohort (*p* < 0.00001).

**Conclusions:**

The study observes that the RA was an independent predictor of DKD in DM patients. The RA, a biomarker that is cost‐effective and easy‐to‐access, may have potential for risk stratification of DKD.

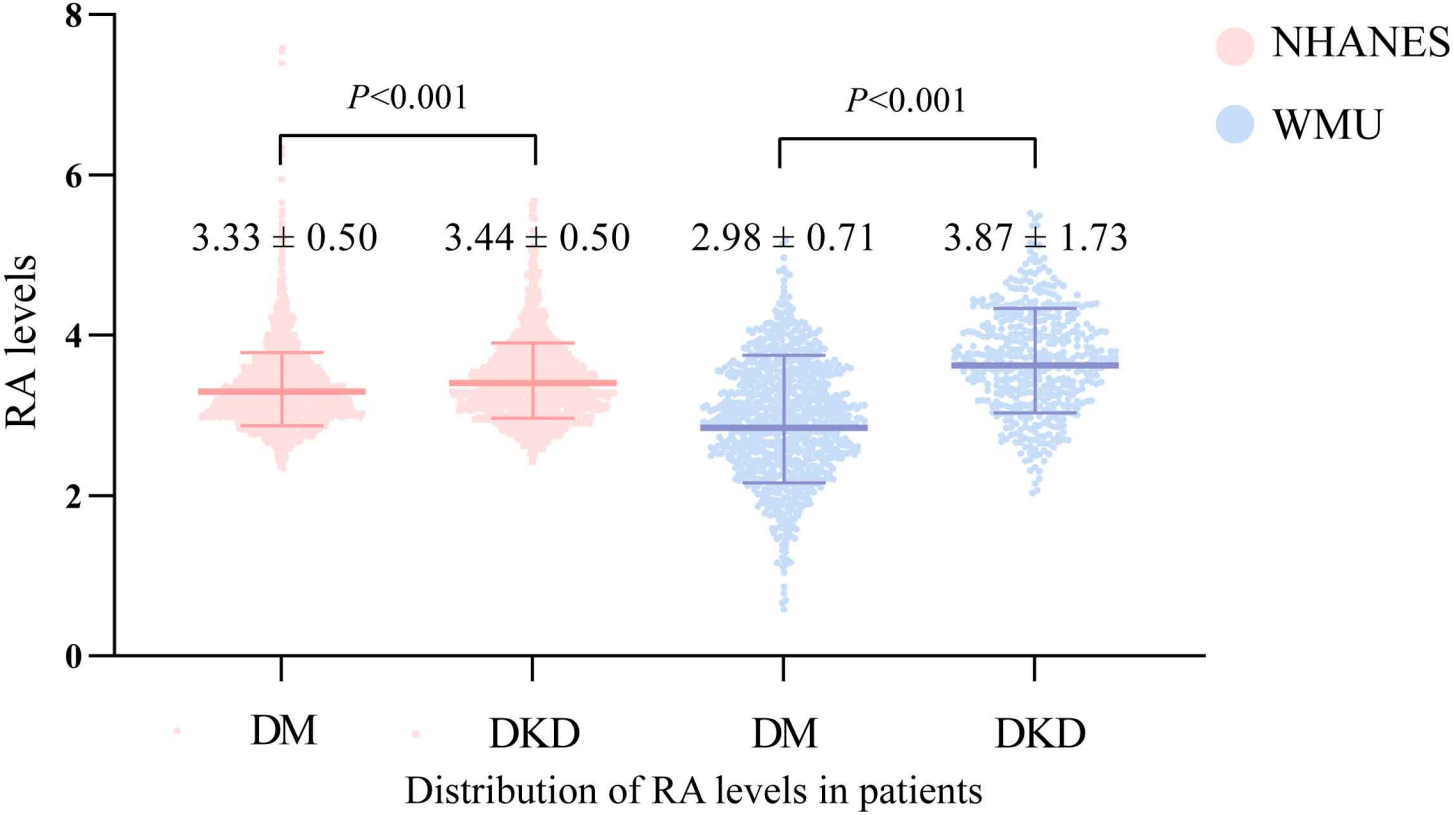

## Graphical abstract

Highlights

- RA levels differ between DM and DKD patients in both the NHANES databases and WMU data.
- After accounting for confounders and using propensity score‐matched analysis, both RA levels were found to be independently associated with DKD.
- The RA, a cost‐effective and easily accessible biomarker, could be useful for risk stratification of DKD.

## 1 INTRODUCTION

Diabetes mellitus (DM) is a heterogeneous illness with etiology hallmarked by hyperglycemia. The global diabetic population is expected to reach 439 million by the year 2030.^1^, ^2^, ^3^ Because of the dramatic increase in diabetes occurrences globally, diabetic kidney disease (DKD), a high‐morbidity microvessel complication of diabetes, has turned into a significant contributor to end‐stage renal disease.^3^, ^4^ DKD has been a source of concern among practitioners for decades as a costly and persistent complication associated with an increased risk of renal development and severe cardiovascular and mortality outcomes.^5^ Approaches to predicting mortality and progression to ESRD have been studied extensively and now depend primarily on clinical indicators such as certain tubular markers, diabetes duration, proteinuria, glycated hemoglobin A1c (HbA1c), blood pressure, and estimated glomerular filtration rate (eGFR).^6^ Nevertheless, there is a strong necessity to identify alternative invasive and routinely detectable clinical indicators of DKD advancement. In people with diabetes, studies revealed a causative relationship of sterile low‐grade inflammation with DKD. Inflammatory indicators might help diabetic patients predict the onset of DKD.^7^

The aberrant elevations in the levels of inflammatory blood cell markers, including platelet‐to‐albumin ratio (PAR),^8^ neutrophil‐to‐albumin ratio (NAR),^9^ and platelet‐to‐lymphocyte ratio (PLR),^10^ act as useful inflammatory indicators; all markers have been assessed for the ability to predict DKD. However, these biomarkers have limited sensitivity and specificity also probably limited the use of those.

The red cell distribution width (RDW)/serum albumin ratio (RA) is an integrative and new inflammatory marker,^11^, ^12^, ^13^, ^14^ which is based on red cell distributing width and serum albumin. However, the functions of RA and DKD are yet to be clarified. To determine the significance of RA in predicting DKD onset and progression, we conducted cross‐sectional research to examine the link between diabetic RA and DKD while controlling for a wide variety of possible confounders.

## 2 METHOD

### 2.1 Source of data and sample

The NHANES (https://wwwn.cdc.gov/nchs/nhanes/Default.aspx) is a program of studies conducted by the National Center for Health Statistics (NCHS), which is part of the Centers for Disease Control and Prevention in the United States. NHANES focuses on assessing the health and nutritional status of adults and children in the United States. NHANES is unique in that it combines interviews and physical examinations. Approximately 5000 individuals are surveyed each year in this nationally representative survey, making it one of the most comprehensive sources of health information. NHANES has been approved by the Ethics Review Committee of the National Center for Health Statistics and Research. The cross‐sectional study analyzed NHANES data available from 2009 to 2018 prepandemic. According to the questionnaire data DIQ010–Doctor told you have diabetes, we screened out patients with DM and further excluded patients with heart failure, with previous failing kidneys, loss of eGFR or urinary protein creatinine ratio, serum albumin, and RDW data, and drugs that influence the RDW and albumin.

The Ethics Committee at The Second Affiliated Hospital and Yuying Children's Hospital, Wenzhou Medical University, granted its approval for the research. Between January 2019 and December 2021, an aggregate of 1412 diabetes mellitus type 2 (T2DM) patients were evaluated retrospectively. The American Diabetes Association^15^ set the criteria used for T2DM diagnosis. The following were the conditions for exclusion: (1) the presence of other comorbid renal disorders, including antiglomerular basement membrane glomerulonephritis, immunoglobulin A (IgA) nephropathy, and membranous nephropathy; (2) concurrent autoimmune illnesses; (3) heart failure; (4) acute infections or inflammation; and (5) drugs that influence the RDW and albumin.

### 2.2 Assessment of DKD and study variables

DKD was defined as the presence of albumin creatinine ratio and/or eGFR <60 mL/min/1.73 m^2^ in T2DM patients according to the diagnostic criteria from kidney disease outcomes quality initiative clinical practice guideline.^16^

We used the previously proposed equation to calculate RA: RDW (%) divided by albumin concentration (g/dL). Demographic characteristics included age, sex, duration of DM, body mass index, systolic blood pressure, diastolic blood pressure (DBP), HbA1c, eGFR, RDW, serum albumin, stroke, and coronary heart disease (CHD).

### 2.3 Statistical analyses

Continuous variables are expressed as means ± standard deviations (SDs), and categorical variables are expressed as numeric values (%). Statistical comparisons were carried out using the two‐tailed *t*‐test or Mann–Whitney *U*‐test in the case of continuous data, whereas χ^2^ test was used in the case of categorical data. The correlation between RA and DKD was investigated using a multivariate logistic regression analysis. Model 1 did not correct for any possible confounders; model 2 corrected for age, sex, glycated HbA1c, systolic blood pressure, DBP, fasting glucose, chronic conditions including stroke (yes/no), and CHD (yes/no). Model 3 matched age, sex, duration, body mass index, systolic blood pressure, diastolic blood pressure, fasting glucose, glycated HbA1c, total cholesterol (TC), triglyceride (TG), uric acid, creatinine, insulin use, coronary heart disease, and stroke.

Propensity score matching (PSM) was undertaken to eliminate potential bias. PSM was conducted at a ratio of 1:1 using a caliper width of 0.01 of the SD of the logit of the propensity score. R software (version 4.00, The R Foundation) was used for statistical analysis. A two‐sided *p* < 0.05 was considered statistically significant.

## 3 RESULTS

### 3.1 Subject features

A total of 2839 DM patients who satisfied our eligibility requirements were enrolled from the NHANES cohort (1365 females and 1474 males; mean age: 60.82; median RA: 3.3), and 1412 patients with DM were enrolled from the WMU cohort (663 females and 749 males; mean age: 63; median RA: 3.2); 974 patients were diagnosed with DKD in NHANES and 462 in WMU.

DKD patients were mostly older, male, and had higher levels of RDW, RA, UA, fasting glucose, HbA1c, creatinine, and higher instances of CHD and stroke, but lower level of eGFR, albumin, as compared with those without DKD (*p* < 0.05) both in the NHANES and the WMU cohorts (Tables 1 and 2). The distribution of T and TG did not differ in the NHANES cohort, and DBP, TC, and TG did not differ in the WMU cohort.

**TABLE 1:** Characteristics of the study patients in NHANES.

	Diabetes mellitus (*n* = 1865)	DKD (*n* = 974)	*p* value
Age (years)	58.44 ± 12.44	65.39 ± 12.75	<0.001
Male	940 (50.4)	534 (54.8)	0.025
Duration (years)	3.63 ± 75.01	1.99 ± 104.29	<0.001
Body mass index (kg/m^2^)	32.80 ± 7.44	30.86 ± 7.12	<0.001
RA	3.33 ± 0.50	3.44 ± 0.50	<0.001
RDW (%)	13.72 ± 1.44	13.84 ± 1.29	<0.001
Albumin (g/dL)	4.15 ± 0.33	4.07 ± 0.37	<0.001
Systolic blood pressure (mm Hg)	127.96 ± 16.81	138.21 ± 21.78	<0.001
Diastolic blood pressure (mmHg)	69.87 ± 12.59	68.21 ± 15.89	0.004
Fasting glucose (mg/dL)	8.61 ± 3.25	9.47 ± 4.02	<0.001
HbA1c (%)	7.32 ± 1.66	7.83 ± 2.03	<0.001
TC (mmol/L)	4.68 ± 1.13	4.64 ± 1.20	0.159
TG (mmol/L)	1.59 ± 0.96	1.83 ± 1.86	0.395
UA (μmol/L)	324.79 ± 85.09	347.64 ± 91.23	<0.001
Crea (μmol/L)	74.59 ± 19.51	94.35 ± 41.30	<0.001
eGFR (mL/min/1.73 m^2^)	118.59 ± 47.61	90.76 ± 54.36	<0.001
Insulin use, *n* (%)			<0.001
No	1463 (78.5)	667 (68.5)	
Yes	401 (21.5)	307 (31.5)	
Coronary heart disease, *n* (%)			<0.001
No	1751 (94.1)	860 (89.3)	
Yes	109 (5.9)	103 (10.7)	
Stroke, *n* (%)			<0.001
No	1764 (94.7)	866 (89.0)	
Yes	99 (5.3)	107 (11.0)	

Abbreviations: Crea, creatinine; DKD, diabetic kidney disease; eGFR, estimated glomerular filtration rate; HbA1c, glycated hemoglobin A1c; NHANES, National Health and Nutrition Examination Survey; RA, red cell distribution width/serum albumin ratio; RDW, red cell distributing width; TC, total cholesterol; TG, triglyceride; UA, uric acid.

**TABLE 2:** Characteristics of the study patients in WMU.

	Diabetes mellitus (*n* = 950)	DKD (*n* = 462)	*p* value
Age (years)	60.24 ± 10.01	68.91 ± 12.41	<0.001
Male	353 (37.2)	396 (85.7)	<0.001
Duration (years)	8.03 ± 2.03	9.87 ± 1.86	<0.001
Body mass index (kg/m^2^)	23.78 ± 4.98	26.52 ± 5.25	<0.001
RA	2.98 ± 0.71	3.87 ± 1.73	<0.001
RDW (%)	11.78 ± 1.61	14.07 ± 1.91	<0.001
Albumin (g/dL)	4.14 ± 0.67	3.87 ± 0.50	<0.001
Systolic blood pressure (mmHg)	115.20 ± 16.91	145.17 ± 16.92	<0.001
Diastolic blood pressure (mmHg)	59.93 ± 10.26	60.74 ± 9.79	0.050
Fasting glucose (mg/dL)	9.97 ± 2.94	10.86 ± 2.88	<0.001
HbA1c (%)	8.98 ± 3.03	9.91 ± 3.04	<0.001
TC (mmol/L)	4.03 ± 1.15	4.05 ± 1.12	0.543
TG (mmol/L)	1.42 ± 0.50	1.41 ± 0.48	0.873
UA (μmol/L)	249.54 ± 65.33	283.49 ± 65.73	<0.001
Crea (μmol/L)	58.84 ± 19.51	82.17 ± 19.81	0.001
eGFR (mL/min/1.73 m^2^)	90.26 ± 19.89	63.00 ± 19.29	<0.001
Insulin use, *n* (%)			<0.001
No	721 (75.9)	213 (46.1)	
Yes	229 (24.1)	249 (53.9)	
Coronary heart disease, *n* (%)			<0.001
No	690 (72.6)	233 (50.4)	
Yes	260 (27.4)	229 (49.6)	
Stroke, *n* (%)			<0.001
No	785 (82.6)	240 (51.9)	
Yes	165 (17.4)	222 (48.1)	

Abbreviations: Crea, creatinine; eGFR, estimated glomerular filtration rate; RA, red cell distribution width/serum albumin ratio; RDW, red cell distributing width; TG, triglyceride; UA, uric acid; DKD, diabetic kidney disease; HbA1c, glycated hemoglobin A1c; TC, total cholesterol; WMU, Wenzhou Medical University.

The baseline analysis of other chronic inflammation indicators used in the study is given in Table S1. The results show that DKD patients have higher levels of PLR, PAP, and NAR, as well as higher levels of neutrophil and platelet counts. There is no difference in the lymphocyte count between the two groups.

### 3.2 RA independently served as a risk factor for DKD

For the NHANES cohort, the effect sizes (odds ratio [OR]) and 95% confidence intervals (CIs) are depicted in Table 3. RA ≥3.3 was a risk factor for DKD (OR = 1.461, 95% CI: 1.250–1.707, *p* < 0.00001). After adjusting for age, sex, HbA1c, SBP, DBP, fasting glucose, chronic conditions including stroke (yes/no) and CHD (yes/no), RA ≥3.3 independently served as a risk indicator for DKD (OR = 1.409, 95% CI: 1.086–1.828, *p* = 0.00994). Similar results were observed in the WMU cohort (Table 4). Higher RA (≥3.2) and RAR levels were significantly associated with a higher risk of DKD (OR = 8.700, 95% CI: 6.624–11.426, *p* < 0.00001). This association remained statistically significant adjusting for confounders (OR = 8.417, 95% CI: 5.042–14.051, *p* < 0.00001). When divided into three and four groups based on RA levels, the results were consistent with those observed in the two‐group model (*p* < 0.05; Tables S2 and S3).

**TABLE 3:** Association between RA and DKD in patients of NHANES.

RA	OR	95% CI	*p* value
Before propensity score			
< 3.3	Reference		
≥3.3	1.461	1.250–1.707	<0.00001
Before propensity score^a^			
<3.3	Reference		
≥3.3	1.409	1.086–1.828	0.00994
After propensity score			
<3.3	Reference		
≥3.3	1.363	1.032–1.800	0.02889

Abbreviations: CHD, coronary heart disease; CI, confidence interval; DBP, diastolic blood pressure; DKD, diabetic kidney disease; HbA1c, glycated hemoglobin A1c; NHANES, National Health and Nutrition Examination Survey; OR, odds ratio; SBP, systolic blood pressure.

^a^ Adjusted for age, sex, HbA1c, SBP, DBP, fasting glucose, chronic conditions including stroke (yes/no), CHD (yes/no).

**TABLE 4:** Association between RA and DKD in patients of WMU.

RA	OR	95% CI	*p* value
Before propensity score			
<3.2	Reference		
≥3.2	8.700	6.624–11.426	<0.00001
Before propensity score^a^			
<3.2	Reference		
≥3.2	8.417	5.042–14.051	<0.00001
After propensity score			
<3.2	Reference		
≥3.2	2.138	1.570–2.911	<0.00001

Abbreviations: CHD, coronary heart disease; CI, confidence interval; DBP, diastolic blood pressure; DKD, diabetic kidney disease; HbA1c, glycated hemoglobin A1c; OR, odds ratio; RA, red cell distribution width‐serum albumin ratio; SBP, systolic blood pressure; WMU, Wenzhou Medical University.

^a^ Adjusted for age, sex, HbA1c, SBP, DBP, fasting glucose, and chronic conditions including stroke (yes/no) and CHD (yes/no).

### 3.3 Propensity score‐matched analysis

PSM was undertaken to further comprehend the relationship of RA with DKD. In the NHANES cohort, patients in distinct RA groups did not have different baseline features except for BMI (Table 5). In logistic regression analysis, elevated RA levels (RA ≥3.3) were independently linked to DKD (OR = 1.363, 95% CI: 1.032–1.800, *p* = 0.02889). This result in the WMU cohort is similar to the NHANES cohort (Table 6). Patients in distinct RA groups did not have substantially different baseline features. Higher RA (≥3.2) levels were significantly associated with a higher risk of DKD (OR = 2.138, 95% CI: 1.570–2.911, *p* < 0.00001).

**TABLE 5:** Characteristics of patients before and after PSM in NHANES.

	Before propensity score		After propensity score	
	<3.3	≥3.3	<3.3	≥3.3
	(*n* = 1479)	(*n* = 1360)	(*n* = 469)	(*n* = 469)
Age (years)	59.92 ± 12.70	61.80 ± 13.20*	61.50 ± 11.93	61.17 ± 13.03
Male	880 (59.5)	594 (43.6)*	257 (54.8)	232 (49.5)
Duration (years)	3.12 ± 78.77	3.01 ± 93.58*	3.45 ± 73.72	5.93 ± 76.02
BMI (kg/m^2^)	30.44 ± 6.06	34.00 ± 8.21*	30.93 ± 6.08	32.32 ± 7.04*
SBP (mmHg)	130.50 ± 18.42	132.40 ± 20.08*	131.48 ± 18.96	131.65 ± 19.22
DBP (mmHg)	70.09 ± 13.28	68.45 ± 14.32*	70.07 ± 13.70	69.27 ± 14.25
Fasting glucose (mmol/L)	8.82 ± 3.42	8.98 ± 3.69	8.85 ± 3.36	8.88 ± 3.79
HbA1c (%)	7.47 ± 1.80	7.52 ± 1.83	7.53 ± 1.85	7.50 ± 1.90
TC (mmol/L)	4.73 ± 1.17	4.60 ± 1.14*	4.62 ± 1.08	4.58 ± 1.11
TG (mmol/L)	1.73 ± 1.47	1.60 ± 1.17	1.66 ± 1.06	1.61 ± 1.23
UA (μmol/L)	327.39 ± 83.70	338.32 ± 91.94*	334.70 ± 86.92	333.74 ± 88.29
Crea (μmol/L)	78.60 ± 22.64	84.38 ± 36.77*	78.04 ± 23.77	80.09 ± 34.59
eGFR (mL/min/1.73 m^2^)	107.92 ± 45.16	110.93 ± 58.13	107.08 ± 44.76	113.64 ± 55.89*
Insulin use, *n* (%)		*		
Yes	290 (19.6)	418 (30.7)	106 (22.6)	121 (25.8)
No	1188 (80.4)	942 (69.3)	363 (77.4)	348 (74.2)
CHD, *n* (%)		*		
Yes	96 (6.5)	116 (8.6)	39 (8.3)	40 (8.5)
No	1375 (93.5)	1236 (91.4)	430 (91.7)	429 (91.5)
Stroke, *n* (%)		*		
Yes	81 (5.5)	125 (9.2)	25 (5.3)	34 (7.2)
No	1397 (94.5)	1233 (90.8)	444 (94.7)	435 (92.8)
DKD, *n* (%)		*		*
Yes	447 (30.2)	527 (38.8)	130 (27.7)	161 (34.3)
No	1032 (69.8)	833 (61.25)	339 (72.3)	308 (65.7)

*Note*: 1:1 matching for age, sex, duration, BMI, SBP, DBP, fasting glucose, HbA1c, TC, TG, UA, Crea, insulin use, CHD, and stroke.

Abbreviations: BMI, body mass index; CHD, coronary heart disease; Crea, creatinine; DBP, diastolic blood pressure; DKD, diabetic kidney disease; eGFR, estimated glomerular filtration rate; HbA1c, glycated hemoglobin A1c; NHANES, National Health and Nutrition Examination Survey; PSM, propensity score‐matched; SBP, systolic blood pressure; TC, total cholesterol; TG, triglyceride; UA, uric acid.

* *p* < 0.05.

**TABLE 6:** Characteristics of patients before and after PSM in WMU.

	Before propensity score		After propensity score	
	<3.2	≥3.2	<3.2	≥3.2
	(*n* = 706)	(*n* = 706)	(*n* = 465)	(*n* = 465)
Age (years)	61.07 ± 10.48	65.09 ± 12.28*	61.87 ± 10.95	62.65 ± 11.69
Male	298 (42.2)	451 (63.9)*	137 (17.3)	137 (17.3)
Duration (years)	8.25 ± 2.13	9.01 ± 2.11*	8.37 ± 2.11	8.39 ± 2.12
BMI (kg/m^2^)	23.86 ± 4.99	25.49 ± 5.33*	24.06 ± 4.98	24.69 ± 5.12
SBP (mmHg)	118.57 ± 19.61	131.45 ± 22.37*	120.94 ± 20.95	123.25 ± 21.02
DBP (mmHg)	59.98 ± 10.02	60.41 ± 10.21	60.69 ± 9.96	60.15 ± 10.19
Fasting glucose (mmol/L)	10.11 ± 2.94	10.43 ± 2.96*	10.26 ± 2.91	10.23 ± 2.95
HbA1c (%)	9.03 ± 3.00	9.54 ± 3.10*	9.06 ± 3.00	9.33 ± 3.19
TC (mmol/L)	4.08 ± 1.12	4.00 ± 1.16	4.10 ± 1.15	3.99 ± 1.16
TG (mmol/L)	1.40 ± 0.51	1.42 ± 0.47	1.37 ± 0.52	1.40 ± 0.48
UA (μmol/L)	251.29 ± 66.49	269.94 ± 66.97*	250.47 ± 66.82	258.83 ± 66.49
Crea (μmol/L)	61.86 ± 20.77	71.09 ± 23.13*	63.20 ± 21.78	64.63 ± 22.09
eGFR (mL/min/1.73 m^2^)	87.78 ± 21.93	74.90 ± 23.23*	85.95 ± 22.95	82.48 ± 22.38
Insulin use, *n* (%)		*		
Yes	200 (28.33)	278 (39.38)	137 (29.5)	142 (30.5)
No	506 (71.67)	428 (60.62)	328 (70.5)	323 (69.5)
CHD, *n* (%)		*		
Yes	219 (31.02)	270 (38.24)	151 (32.5)	145 (31.2)
No	487 (68.98)	436 (61.76)	314 (67.5)	320 (68.8)
Stroke, *n* (%)		*		
Yes	554 (78.47)	235 (33.29)	107 (23)	116 (24.9)
No	152 (21.53)	554 (78.47)	358 (77)	349 (75.1)
DKD, *n* (%)		*		*
Yes	83 (11.76)	379 (53.68)	83 (11.76)	143 (18.1)
No	623 (88.24)	327 (46.32)	383 (82.4)	319 (68.6)

*Note*: 1:1 matching for age, sex, duration, BMI, SBP, DBP, fasting glucose, HbA1c, TC, TG, UA, Crea, insulin use, CHD, stroke.

Abbreviations: BMI, body mass index; CHD, coronary heart disease; Crea, creatinine; DBP, diastolic blood pressure; DKD, diabetic kidney disease; eGFR, estimated glomerular filtration rate; HbA1c, glycated hemoglobin A1c; PSM, propensity score‐matched; SBP, systolic blood pressure; TC, total cholesterol; TG, triglyceride; UA, uric acid; WMU, Wenzhou Medical University.

* *p* < 0.05.

### 3.4 Diagnostic efficacy of various parameters for DKD

**FIGURE 1:**
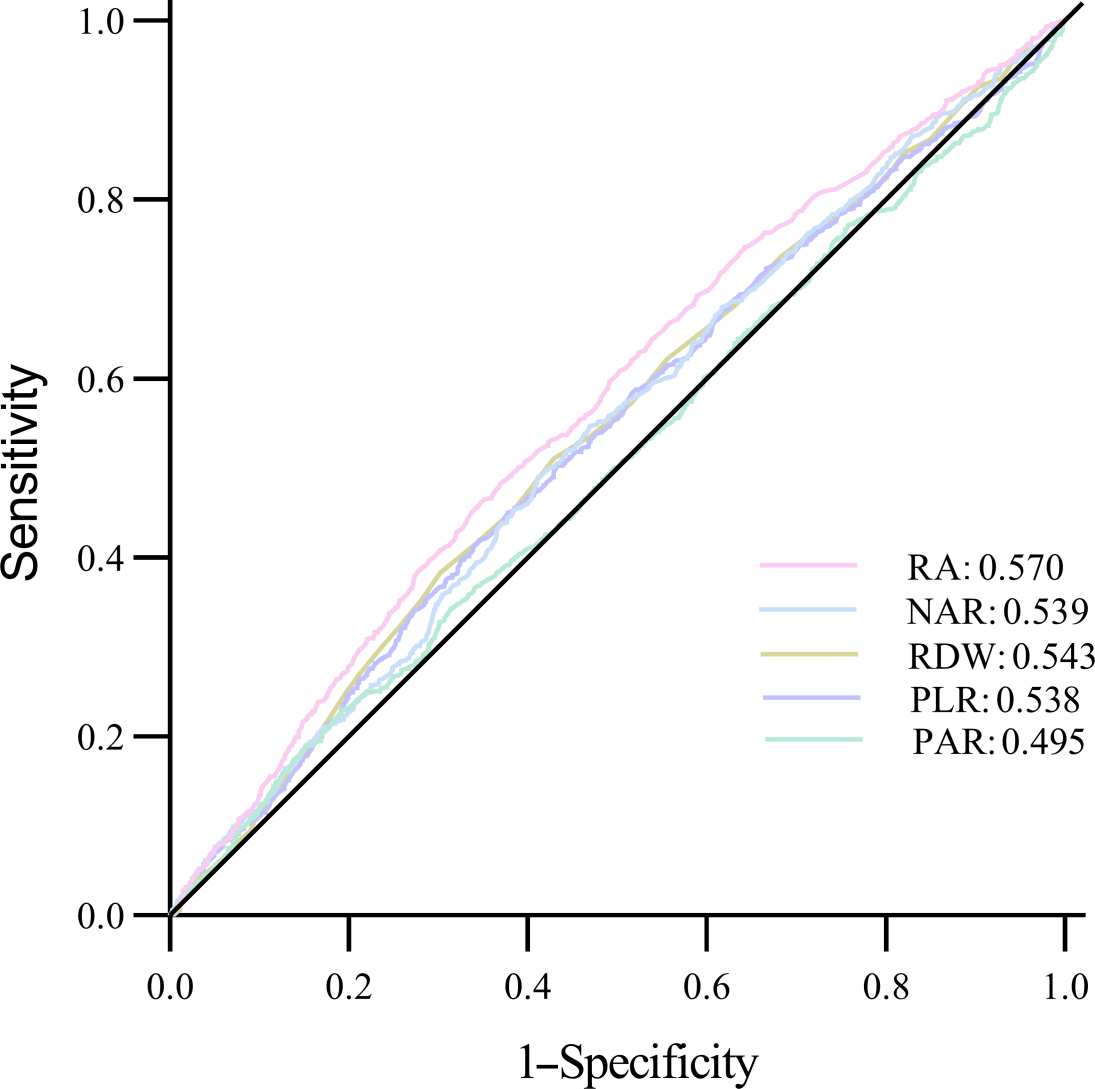
The receiver operating characteristic curves of the RA, RDW, PLR, PAR, and NAR for diabetic kidney disease. The area under curves for RA, NAR, RDW, PLR, and PAR are 0.570, 0.539, 0.543, 0.538, and 0.495, respectively. NAR, neutral to albumin ratio; PAR, platelet to albumin ratio; PLR, platelet to lymphocyte ratio; RA, red cell distribution width/albumin ratio; RDW, red cell distribution width.

To assess the diagnostic effectiveness of the RA, RDW, PLR, PAR, and NAR for DKD, a receiver operating characteristic curve was used (Figure 1). The result displayed that the sensitivity of RA was lower than PLR (0.652 vs 0.848; Table S3), the specificity of RA was lower than RDW and NAR (0.462 vs 0.510, 0.462 vs 0.490; Table S4), but the overall predictive value of RA was better than RDW, PLR, PAR, and NAR in DM patients (*p* < 0.05, Table S5).

## 4 DISCUSSION

To the best of our knowledge, this research is the first to demonstrate the strong link between RA and DKD for people with diabetes. Through two cohorts of data, we observed that patients with DKD showed significantly higher RA than diabetic patients without DKD. Another significant discovery was that a higher RA level independently functioned as a risk indicator for DKD.

The presence of a chronic inflammatory cell milieu predisposes to increased molecular apoptosis and, as a result, promoted red blood cell (RBC) degradation.^17^ The molecular mechanisms associating chronic inflammation with anisocytosis have been hypothesized among diabetic and chronic kidney disease patients including a decrease in erythrocyte lifespan, suppression in erythropoietin response, and impairment in iron metabolism.^18^, ^19^ Moreover, T2DM is also known to be a chronic inflammation‐related disease.^20^, ^21^ Systemic inflammation plays an important role in DKD progression.^22^, ^23^ RA is an integrative and new inflammatory marker,^11^, ^12^, ^13^, ^14^ which is based on RDW and serum albumin.

RDW expresses changes in the size of RBCs and has been identified as a new prognostic factor in many pathophysiological conditions.^24^ RDW was illustrated as having a considerable link to greater c‐reactive protein levels in diabetes patients. RDW reliably predicts mortality and complication incidence in the general populace, according to data from population research. RDW was found to independently function as a predictor for all‐cause and CHD deaths.^25^ RDW could be employed as a new indicator of T2DM onset and advancement, as well as a biomarker of diabetes‐related complications.^26^

The course of treatment to reduce appetite in diabetic patients affects the uptake of nutrients, causing one or more metabolic disorders. Conversely, nutrient deficiency and metabolic disorders will also aggravate the disease, affecting the prognosis of patients, forming a vicious cycle which may lead to lower albumin. Albumin constitutes about 40%–60% of the total plasma proteins^27^, ^28^ and is abundant in plasma where it is strongly associated with the nutritional status. Previous studies have shown that albumin was associated with the development of DKD.^29^

The RA ratio may be a superior tool to other single identified markers in evaluating inflammatory response.^12^, ^30^, ^31^, ^32^ Because the RA ratio is rapidly and easily evaluated using laboratory examinations,^13^, ^33^ it can function as a simple but relatively reliable index for stratification of diabetic patients. To the best of our knowledge, to date, no study has examined the association between RA and the DKD. The strength of this study lies in the elucidation of the complete relationship between RA and the DKD.

Our research, however, has certain shortcomings: (1) Owing to the retrospective observational research method used in this research, a causal relationship cannot be constructed. To remedy this, prospective research is required. (2) Only one blood test was used to extract data. Considering that blood cells have a limited life span, serial testing might be more applicable as opposed to a single test on admission.

## 5 CONCLUSION

In conclusion, we postulate that RA is a novel and independent predictor of DKD in diabetic patients, and the RA may be used to predict DKD. However, extensive prospective and long‐term follow‐up studies are needed to validate the relationship between RA and DKD in diabetic patients.

## AUTHOR CONTRIBUTIONS

Jiaqi Chen wrote the manuscript. Daguan Zhang and Depu Zhou collected the data. Jiaqi Chen and Daguan Zhang analyzed the data. Zhijuan Dai revised the manuscript. Jie Wang designed the study and performed quality control. All authors read and approved the final manuscript.

## FUNDING INFORMATION

The authors did not receive support from any organization for the submitted work.

## CONFLICT OF INTEREST STATEMENT

The authors declare no conflicts of interest.

## Supporting information


**Data S1.** Supporting Information.
