# Supplementary material for: Association between red cell distribution width/serum albumin ratio and diabetic kidney disease
Source: J Diabetes. 2024 Jun 26;16(7):e13575. doi: 10.1111/1753-0407.13575 (PMC11200132; doi:10.1111/1753-0407.13575)
Supplement: Supplementary file 1 — Data S1. Supporting Information. [file JDB-16-e13575-s001.docx]

Supplementary Table S1 Distribution of other chronic inflammation indicators of the study population in Nhanes

|  | Diabetes mellitus | DKD | *p* |
| --- | --- | --- | --- |
| LYMNO | 2.194 ± 0.828 | 2.214 ± 0.960 | 0.859 |
| NENO | 4.339 ± 1.575 | 4.762 ± 1.854 | <0.001 |
| PLT | 229.274 ± 59.601 | 243.299 ± 75.298 | <0.001 |
| PLR | 114.346 ± 43.076 | 123.500 ± 57.638 | <0.001 |
| NAR | 1.006 ± 0.371 | 1.232 ± 0.514 | <0.001 |
| PAR | 53.160 ± 14.344 | 62.919 ± 20.848 | <0.001 |

Abbreviations: LYMNO, lymphocyte count; NENO, neutrophil count; PLT, platelet counts; PLR, platelet-to-lymphocyte ratio; PAR, platelet-to- albumin; NAR, neutrophil-to- albumin

Supplementary Table S2 Association between RA and DKD in patients of Nhanes

| RA | Crude | |  | Adjust | |
| --- | --- | --- | --- | --- | --- |
|  |  |  |  | OR (95%CI) | *p* |
| RA tertile |  |  |  |  |  |
| T1 | Reference |  |  | Reference |  |
| T2 | 1.372 (1.128, 1.670) | 0.00159 |  | 1.631 (1.176, 2.261) | 0.00336 |
| T3 | 1.878 (1.549, 2.277) | <0.00001 |  | 1.896 (1.365, 2.632) | 0.00013 |
|  |  |  |  |  |  |
| RA quartile |  |  |  |  |  |
| Q1 | Reference |  |  | Reference |  |
| Q2 | 1.379 (1.096, 1.734) | 0.00601 |  | 1.644 (1.120, 2.414) | 0.01116 |
| Q3 | 1.442 (1.150, 1.809) | 0.00155 |  | 1.614 (1.099, 2.369) | 0.01460 |
| Q4 | 2.002 (1.601, 2.504) | <0.00001 |  | 2.175 (1.485, 3.186) | 0.00007 |

Crude: no adjustment. Adjust: adjusted for age, sex, HbA1c, SBP, DBP, fasting glucose, chronic conditions including stroke(yes/no), CHD (yes/no).

Supplementary Table S3 Association between RA and DKD in patients of WMU

| RA | Crude | |  | Adjust | |
| --- | --- | --- | --- | --- | --- |
|  |  |  |  | OR (95%CI) | *p* |
| RA tertile |  |  |  |  |  |
| T1 | Reference |  |  | Reference |  |
| T2 | 3.893 (2.724, 5.564) | <0.00001 |  | 3.276 (2.079, 5.160) | <0.00001 |
| T3 | 11.838 (8.343, 16.797) | <0.00001 |  | 10.471 (6.657, 16.470) | <0.00001 |
|  |  |  |  |  |  |
| RA quartile |  |  |  |  |  |
| Q1 | Reference |  |  | Reference |  |
| Q2 | 4.625 (2.814, 7.601) | <0.00001 |  | 4.137 (2.265, 7.557) | <0.00001 |
| Q3 | 10.007 (6.184, 16.191) | <0.00001 |  | 8.809 (4.845, 16.016) | <0.00001 |
| Q4 | 23.721 (14.653, 38.401) | <0.00001 |  | 21.647 (11.835, 39.595) | <0.00001 |

Crude: no adjustment. Adjust: adjusted for age, sex, HbA1c, SBP, DBP, fasting glucose, chronic conditions including stroke(yes/no), CHD (yes/no).

Supplementary Table S4 The ROC analysis of the RA, RDW, PLR, PAR and NAR for DKD

|  | Sensitivity | Specificity | AUC |
| --- | --- | --- | --- |
| RA | 0.652 | 0.462 | 0.570 |
| RDW | 0.571 | 0.510 | 0.543 |
| PLR | 0.652 | 0.421 | 0.538 |
| PAR | 0.848 | 0.188 | 0.495 |
| NAR | 0.589 | 0.490 | 0.539 |

Abbreviations: RA, red cell distributing width- albumin ratio; RDW, red cell distributing width; PLR, platelet-to-lymphocyte ratio; PAR, platelet-to- albumin; NAR, neutrophil-to- albumin

Supplementary Table S5 The different of AUC area of the RA with RDW, PLR, PAR and NAR for DKD.

|  | *p* value |
| --- | --- |
| RA-RDW | 0.001 |
| RA-PLR | 0.034 |
| RA-PAR | <0.001 |
| RA-NAR | 0.021 |

Abbreviations: RA, red cell distributing width- albumin ratio; RDW, red cell distributing width; PLR, platelet-to-lymphocyte ratio; PAR, platelet-to- albumin; NAR, neutrophil-to- albumin
